# Dual Functions of Androgen Receptor Overexpression in Triple-Negative Breast Cancer: A Complex Prognostic Marker

**DOI:** 10.3390/bioengineering12010054

**Published:** 2025-01-10

**Authors:** Umay Kiraz, Emma Rewcastle, Silja K. Fykse, Ingrid Lundal, Einar G. Gudlaugsson, Ivar Skaland, Håvard Søiland, Jan P. A. Baak, Emiel A. M. Janssen

**Affiliations:** 1 Department of Pathology, Stavanger University Hospital, 4011 Stavanger, Norway; emma.rewcastle@sus.no (E.R.); silja.kavlie.fykse@sus.no (S.K.F.); ingrid.lundal@sus.no (I.L.); einar.gudbjorn.gudlaugsson@sus.no (E.G.G.); ivar.skaland@sus.no (I.S.); jpabaak47@yahoo.com (J.P.A.B.); emilius.adrianus.maria.janssen@sus.no (E.A.M.J.); 2 Department of Chemistry, Bioscience and Environmental Engineering, University of Stavanger, 4021 Stavanger, Norway; 3 Department of Research, Stavanger University Hospital, 4011 Stavanger, Norway; 4 Institute for Biomedicine and Glycomics, Griffith University, Queensland, QLD 4215, Australia

**Keywords:** triple-negative breast neoplasm, androgen receptor, tumor-infiltrating lymphocytes, fibrotic focus, biomarkers

## Abstract

A subset of triple-negative breast cancer (TNBC) expresses the androgen receptor (AR), but thresholds for AR positivity and its clinical significance vary. We hypothesize that objective assessment outperforms subjective methods, and that high AR negatively impacts prognosis. In a population-based TNBC cohort (*n* = 198) with long follow-up (4–383 months), AR expression was evaluated via subjective scoring (AR-Manual) and automated digital image analysis (AR-DIA). A 10% cut-off value via AR-DIA was the strongest negative prognostic threshold for distant metastases (*p* = 0.008). High AR-DIA correlated with lower grade (*p* = 0.014), and lower proliferation (*p* = 0.004) but also with larger tumors (*p* = 0.047), distant metastasis (*p* = 0.052), and lymph node (LN) positivity (*p* < 0.001), highlighting its dual roles. Multivariate analysis revealed interaction between LN status and AR-DIA (*p* < 0.001) as the strongest prognostic factor, followed by fibrotic focus (FF; *p* = 0.009), mitotic activity index (MAI; *p* = 0.018), and stromal tumor-infiltrating lymphocytes (sTILs; *p* = 0.041). AR-DIA had no additional prognostic value in favorable subgroups but was significant in unfavorable subgroups. In high AR-DIA patients with unfavorable characteristics, ACT did not improve survival, and patients may benefit from AR-targeted therapy. Overall, the DIA method provides reproducibility, high AR-DIA (≥10%) shows opposing survival effects in different TNBC subgroups, and AR evaluation is crucial for prognosis and AR-targeted therapies.

## 1. Introduction

Triple-negative breast cancer (TNBC) is characterized by the absence of estrogen receptor (ER), progesterone receptor (PR), and HER2 [1,2,3], and is a histologically and prognostically heterogeneous disease [4,5,6]. As anti-hormonal therapy and trastuzumab are not an option, chemotherapy is still the preferred treatment modality for TNBC. However, the efficacy of chemotherapy in TNBC is variable, and its associated side effects are significant.

Androgen receptor (AR) is a nuclear steroid hormone receptor that is frequently expressed in breast cancer, particularly in ER-positive cases [7,8], where it is often considered a favorable prognostic marker [9,10]. AR expression is also observed in TNBC cases [11,12,13,14,15,16,17,18], although its prognostic role remains controversial. Some studies have suggested that AR expression in TNBC is associated with a better prognosis [13,16,18,19], while others reported that AR expression is correlated with a worse prognosis [15,18,20], and some reported no correlation at all [17,18,21,22]. In a multi-institutional study, 104 AR-positive (≥1%) TNBC patients from Norway had poorer overall survival than AR-negative TNBC patients [18]. However, an older TNBC study from Norway (37 patients) found no prognostic association with AR expression at a 10% cut-off level [17].

TNBC classification based on AR expression may offer a potential avenue for refining prognosis and identifying new therapeutic targets. At present, treatment options for this aggressive cancer subtype are limited, which again highlights the need for further research to address new treatment strategies. As AR is currently under investigation in TNBC, understanding its relationship with other clinicopathologic characteristics and its role in prognosis and progression is critical. Few studies have comprehensively compared the clinicopathological features of TNBC and AR expression [15,20,23]. Moreover, the lack of standardized protocols for immunohistochemistry (IHC) and AR expression measurement is a significant limitation in studies on AR expression in TNBC. This inconsistency has led to considerable variation in the AR expression reported in different TNBC populations and may also explain the absence of consensus on the thresholds used for prognosis. Rigorously standardized IHC methods that yield reproducible results alongside objective automated digital image analysis (DIA) may improve AR expression evaluation in TNBC. It is well known that DIA methods can minimize observer variability and provide results that are more reproducible, consistent, and objective [24].

In the following study, a large population-based cohort consisting of 198 primary TNBC tumors from a single center in Norway was used, with a very long follow-up. The study aims to test the hypotheses that the objective assessment of AR by means of DIA is superior to the subjective assessment performed manually and that AR has a negative impact on prognosis. The relationships between AR expression and demographic/clinicopathological features were investigated, in addition to its association with distant metastasis-free survival (DMFS). This study shows the importance of determining AR in TNBC for both prognosis and choosing patients for AR-targeted therapies.

## 2. Materials and Methods

This was a non-interventional, retrospective, observational study, as the medicinal products were prescribed in the usual manner by the terms of marketing authorization, and we observed the effects of risk factors, treatments, or other interventions without attempting to determine who is or is not exposed to them [25]. The study was approved by the Norwegian Regional Committee for Medical and Health Research Ethics (REC, 2010/1241). Following REC approval, we did not need to obtain informed consent as the tissue samples had already been removed for diagnostic and treatment purposes. All patients were treated following the Norwegian Breast Cancer Group (NBCG) national guidelines at the time of diagnosis. The patients’ demographic information, including their date of birth, primary diagnosis date, date of first metastasis, treatment modality, status at follow-up, final follow-up date, and date of death, were obtained from the relevant hospital information systems.

### 2.1. Patients and Histopathological Analysis

A total of 284 consecutive TNBC patients diagnosed between 1978 and 2004 at the Stavanger University Hospital (SUH) in Norway were initially considered. The following cases were excluded from the study: in 29 patients, axillary lymph node excision was not performed; 15 patients had contralateral breast cancer (BC); in 20 patients, there was not sufficient tissue for AR staining; and in 22 patients, paraffin blocks were lost. As a result, 198 patients were included in the study. Surgically excised tissue was fixed in 4% buffered formaldehyde, embedded in paraffin, cut at 3 μm, and stained with Hematoxylin and Eosin. Two expert breast pathologists (U.K. and E.G.) evaluated these histopathological specimens. The most representative slide (with the highest grade) was selected and evaluated for each patient. The histological type and grade of the tumor were evaluated according to the World Health Organization (WHO) 2019 classification [26], and fibrotic focus (FF) (present/absent) was defined according to Hasebe et al. [27]. Mitotic activity index (MAI) was assessed according to the WHO classification: counting mitotic count per mm^2^ [26,28] and in 10 high-power fields with a field diameter of 0.42 mm for tumor grading. MAI was divided into two groups: <5 versus ≥5 (MAI5) and <10 versus ≥10 (MAI10). Stromal tumor-infiltrating lymphocytes (sTILs) were evaluated according to the international tumor-infiltrating lymphocyte (TIL) working group guidelines [29] and the cut-off value of sTILs <40% versus ≥40% (sTILs40) was used. ER, PR, and HER2 were assessed for the purposes of this study with adequate quality controls [30]. Only patients with <1% ER, <10% PR, and HER2-negative (0 and 1+ with IHC) status were included in the dataset [26,31].

### 2.2. AR Immunohistochemistry (IHC)

The present study used a rabbit immunoglobulin G1 monoclonal antihuman AR primary antibody (clone: SP107, Ventana Medical Systems, Roche Tissue Diagnostics, Tucson, AZ, USA) at a dilution of 1:50. Dako EnVision Flex+ (Agilent Technologies, Santa Clara, CA, USA) was used as the detection system. An automatic IHC staining device, Dako Omnis (Agilent Technologies, Santa Clara, CA, USA), was used. This antibody has been used in several previous BC studies [32,33,34]. Appropriate negative and positive controls were used during staining.

### 2.3. Assessment of IHC Staining

**Figure 1:**
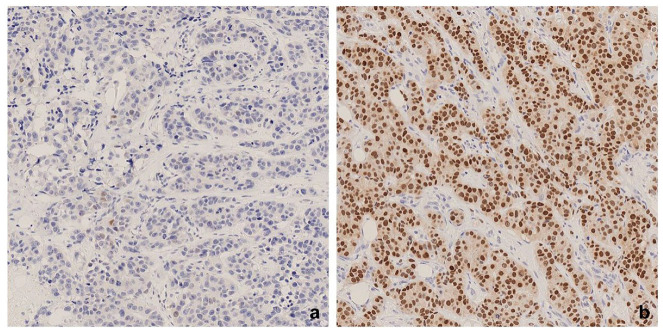
Androgen receptor (AR) expression; low AR (<10%) (**a**) and high AR (≥10%) (**b**).

TNBC samples were globally scored as a continuous variable as a percentage of positive tumor nuclei. AR positivity (high AR) was defined as a nuclear AR expression in ≥10% of tumor cells, while samples with AR expression in <10% of tumor cells were considered low AR (Figure 1). Whole sections stained with AR were scanned at 40x magnification using the Hamamatsu Nanozoomer S60 (Hamamatsu Photonics, Hamamatsu City, Japan) at SUH.

Manual method (AR-Manual): Global AR staining expression was independently scored by U.K. and H.S., who evaluated whole slide images (WSIs) on a computer screen. They were blinded to clinical information and results from other biomarkers. In cases of disagreement, consensus was reached after discussion. U.K. re-assessed randomly selected cases (*n* = 50) to evaluate the reproducibility of the method.

**Figure 2:**
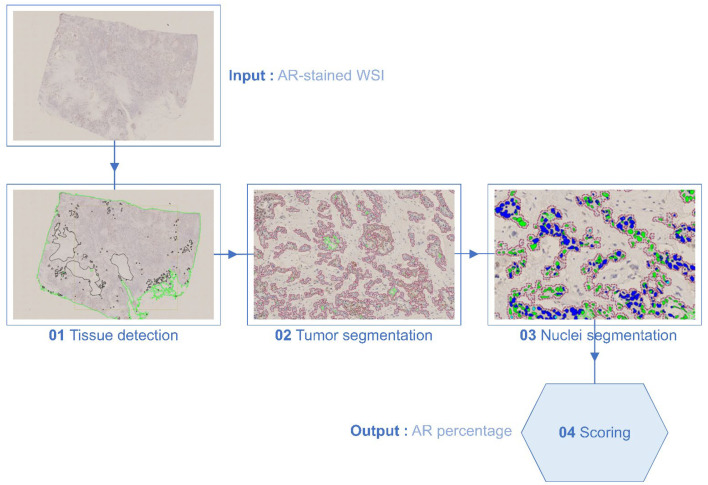
AR-DIA workflow for assessment of androgen receptor percentage. The workflow is divided into four stages: 01: tissue detection (tissue defined by green); 02: tumor segmentation (tumor defined by red); 03: nuclei segmentation (blue: AR-stained cells, green: non-stained cells); and 04: scoring.

Digital image analysis (DIA): An in-house APP (AR-DIA) was developed using the Visiopharm (version 2022.09.3.12885, Visiopharm A/S, Hørsholm, Denmark) platform for global scoring of AR on WSI. A training set, which consisted of four WSIs of good quality and representative of AR positivity, was selected and used to create manual annotations to train classification algorithms. Manually annotated labels of representative regions were made to train the classifier APPs—tissue and background (APP_01), tumor and non-tumor (APP_02), and positive brown and negative blue nuclei (APP_03). This training continued until the results were considered satisfactory. All classifier APPs used Bayesian methods. Additional post-processing steps were added to further enhance segmentation. A global score was generated for the in-house APP (APP_04). Four separate APPs were developed and run in tandem. The runtime of the batch was between 3 and 10 minutes. The APPs consisted of the following: 01 tissue detection, 02 tumor segmentation, 03 nuclei segmentation, and 04 scoring (Figure 2).

### 2.4. Survival Endpoints

For survival analyses, distant metastasis-free survival (DMFS) was used as the endpoint, defined as any recurrence at a distant site or death from distant TNBC metastases (*n* = 69). All other patients were censored, from the date of the last follow-up visit, either as being alive and well (*n* = 96) or as dead from other causes than BC (*n* = 28) or local/regional recurrence (*n* = 5). Due to the limited number of cases with distant metastasis after 15 years (*n* = 2), we used both long-term follow-up data and data from the first 15 years in the survival analysis. Any follow-up periods longer than 15 years were capped at 15 years. The patient’s age, time to first distant metastasis, and follow-up time were computed based on the date of primary diagnosis.

### 2.5. Statistical Analysis

Statistical analyses were performed using software packages from SPSS (version 26.0; IBM SPSS Statistics, Armonk, NY, USA) and MedCalc (version 22, MedCalc Ltd., Ostend, Belgium). A probability of rejecting or failing to reject the null hypothesis (*p*-value) of less than 0.05 was regarded as statistically significant. The Spearman correlation coefficient (rho coefficient) test was used to determine the agreement between continuous variables that were not normally distributed. Receiver operating characteristic (ROC) curve analysis, medians, and quartiles were used to establish the optimal prognostic thresholds of continuous variables. The chi-squared test was used to evaluate the relationship between grouped variables. Additionally, odds ratio (OR) and its confidence interval (CI) were calculated using the chi-squared test. Kaplan–Meier analysis was used to construct survival curves. The relative importance of potential prognostic factors was analyzed using Cox regression analysis and reported as a hazard ratio (HR) with 95% CI.

## 3. Results

Of the 198 TNBC patients, 69 (35%) developed distant metastasis. The follow-up period ranged from 4 to 383 (median: 128) months. The median tumor size, MAI, and sTILs in the 198 TNBC cases were 2.20 cm (range 0.5–10 cm), 16 mitoses per mm^2^ (range 0–131), and 10% (range 1–99%), respectively. FF was observed in 90 patients (46%), and lymph node (LN) positivity in 95 patients (48%).

### 3.1. AR Expression

**Figure 3:**
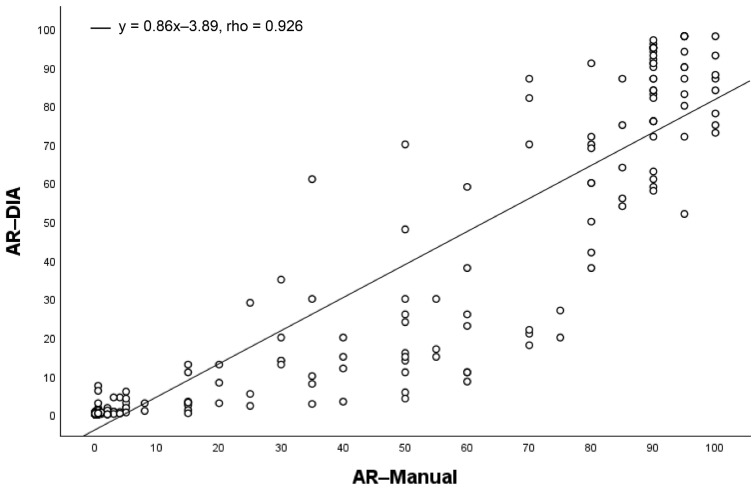
The scatter plot illustrates the relationship between manual counting (AR-Manual), which is shown on the *x*-axis, and digital image analysis (AR-DIA), which is shown on the *y*-axis. The line represents a linear relationship and indicates a strong positive correlation between the variables.

The AR-Manual and AR-DIA measurements were strongly correlated (rho = 0.926, *p* < 0.001), according to the Spearman correlation test. However, manual counting scores showed a trend for higher AR values than DIA scores (Figure 3). The median score was much higher (30%) in the AR-Manual than in AR-DIA (11%) (Figure S1a). The median score of AR-DIA was 23% in LN-positive patients and 3% in LN-negative patients (Figure S1b).

Assessments of different optimal thresholds for AR-Manual and AR-DIA revealed 10% as the strongest prognostic cut-off value for both methods. The sensitivities, specificities, negative and positive predictive values, and overall correct predicted percentages confirmed the value of 10% for AR-DIA (Table S1). Using a 10% cut-off, 59% (117/198) of cases were classified as high AR by AR-Manual, and 51% (101/198) by AR-DIA (Table 1). AR-Manual identified a significantly higher proportion (86%) of cases as ≥10% positive, compared to AR-DIA.

Although the agreement between AR-Manual and AR-DIA methods was reasonable with a 10% cutoff, the preference for subjective manual scoring or DIA quantification may lead to clinically important differences. Comparison of the same observer’s first and second manual evaluations revealed discordance in 6 of 50 cases: 2 with <10% and 4 with ≥10% AR expression.

### 3.2. AR Expression and Clinicopathological Features

Table 2 shows the characteristics of the tumors according to AR-Manual and AR-DIA. AR-DIA ≥ 10% expression was significantly correlated with favorable prognostic characteristics, such as lower tumor grade (1 vs. 2 vs. 3, *p* = 0.038; 1, 2 vs. 3, *p* = 0.014), MAI < 5 (*p* = 0.004), and MAI < 10 (*p* < 0.001). However, it was also correlated with prognostically unfavorable characteristics, including larger tumor size (*p* = 0.047), LN positivity (*p* < 0.001), and distant metastasis (0.052). The correlation between sTILs and AR-DIA was not significant, but there was a trend for high AR patients to have lower sTILs. No significant correlations were found with age, histological type, or FF.

### 3.3. AR Expression and Prognosis

Table 3 shows the univariate survival and hazard ratios of all characteristics concerning DMFS in the first 15 years of follow-up. Tumor grade (1, 2 vs. 3; *p* = 0.029), MAI5 (*p* = 0.015), sTILs40 (*p* = 0.012), FF (*p* = 0.001), AR-DIA (*p* = 0.008), and LN status (*p* < 0.001) were significant univariate outcome predictors. Age, tumor size, tumor grade (1 vs. 2 vs. 3), histologic type, MAI10, AR-Manual, and treatment type were not significant. Similar results in univariate survival analyses were observed in the long-term follow-up (Table S2). Based on these results, only AR-DIA and the 15-year follow-up time were used for further analyses.

A multivariate survival analysis of all univariably significant features revealed that LN status (*p* = 0.001) was the strongest prognostic factor (HR = 2.36, 95% CI 1.41–3.93), followed by FF (*p* = 0.013), AR-DIA (*p* = 0.010), MAI5 (*p* = 0.019), and sTILs40 (*p* = 0.037). Including the interactions between variables, the LN status and AR-DIA interaction became a significant predictor of survival (*p* < 0.001, HR = 1.63, 95% CI 1.34–1.98), indicating that the combined presence of these two factors substantially affects patient outcomes. While the individual effects of LN status (*p* = 0.670) and AR-DIA (*p* = 0.559) were no longer statistically significant, FF (*p* = 0.009), MAI5 (*p* = 0.018), and sTILs40 (*p* = 0.041) remained significant (Table 4).

**Figure 4:**
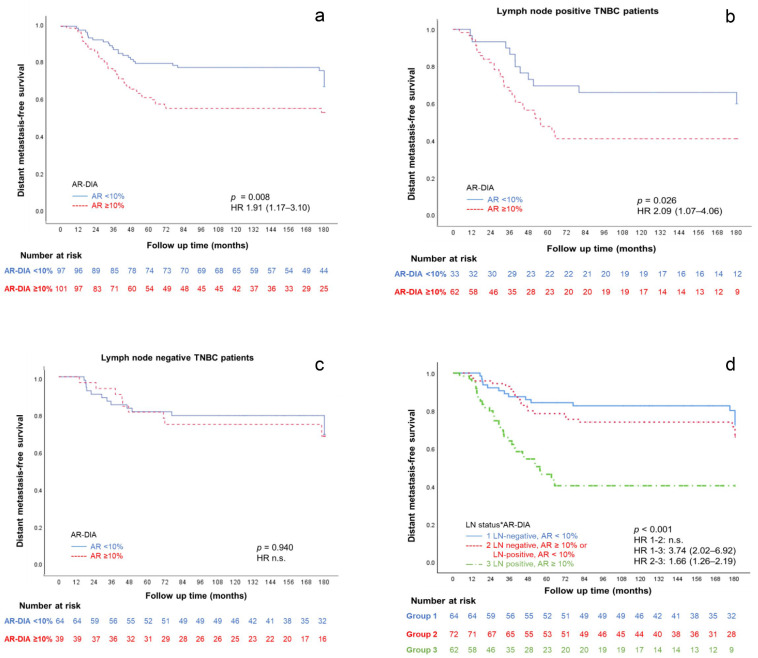
Survival curves of AR-DIA with a 10% threshold in all TNBC cases (**a**), in LN-positive TNBC cases (**b**), in LN-negative TNBC cases (**c**), and survival curves of the interaction between LN status and AR-DIA in all TNBC cases (**d**).

Kaplan–Meier survival curves for the AR-DIA were constructed to visualize DMFS in all cases (Figure 4a), LN-positive cases (Figure 4b), and LN-negative cases (Figure 4c) (*p* = 0.008, *p* = 0.026, and *p* = 0.940, respectively). High AR-DIA was an unfavorable prognostic factor in TNBCs (survival rate 58%) and in LN-positive cases (survival rate 47%), while low AR-DIA was associated with better DFMS in both TNBCs (survival rate 72%) and LN-positive (survival rate 64%) cases. The interaction between LN status and AR-DIA revealed that low AR-DIA in LN-negative patients led to a better survival rate (77%) when compared to high AR-DIA in LN-positive patients (47%) (*p* < 0.001) (Figure 4d).

In prognostically unfavorable subgroup combinations of LN positivity, MAI ≥ 5, FF-present, and sTILs < 40%; high AR-DIA was significantly worsened DMFS (Table 5). Contrary to this, in subgroup combinations of prognostically favorable features, high AR-DIA was rare and did not influence DMFS in any of these subgroups (Kaplan–Meier 0.147 < *p*-value < 0.946).

Adjuvant chemotherapy (ACT) had no significant survival-improving effect in either the overall group of 175 patients (*p* = 0.978), or in the low AR-DIA (84 patients; *p* = 0.409) and high AR-DIA (91 patients; *p* = 0.425) subgroups. However, in exploratory analyses, ACT showed a significant positive effect on prognosis in low AR-DIA patients with different combinations of unfavorable characteristics (LN-positive, MAI ≥ 5, FF-present, and sTILs < 40) (Table S3). In the patients with high AR-DIA with similar combinations of unfavorable characteristics, ACT did not show any survival improvement, and there was a trend suggesting that the non-ACT patients had better survival. Unfortunately, the subgroups with unfavorable characteristics and high AR-DIA included few patients.

AR-DIA was predictive in the ACT subgroup and had a significant effect on survival (*p* = 0.011). In this subgroup, patients with low AR-DIA had a better survival rate (73%), compared to those with high AR-DIA (53%). Conversely, this effect was not observed in the non-ACT group.

## 4. Discussion

The behavior, treatment effects, and prognosis of TNBC differ from those of all other BC subtypes. While AR positivity is often considered a favorable prognostic factor in ER-positive breast cancer patients, its impact on TNBC prognosis has yielded varying results. This may be partly due to the limitations of previous studies, including short follow-up, the uncertainty of threshold, non-standardized IHC procedures, and assessment techniques.

An international consensus on the cut-off values for determining AR negativity/positivity has not yet been developed, and the decision thresholds used in different studies range from 0% to 75% [10,11,15,17,18,19,21,35,36], although the 1% and 10% thresholds are most commonly used [23]. We analyzed different thresholds of AR expression and found that 10% was the strongest prognostic cut-off value. Avoiding different threshold values will only be beneficial if strictly standardized tissue processing and immunohistochemistry protocols are adopted and reproducible AR expression measurement techniques are used.

The AR-Manual and AR-DIA methods demonstrated a strong correlation, although the DIA method showed a stronger prognostic effect and better reproducibility compared to the AR-Manual method. As AR-Manual assessment showed discordance when repeated, it is considered unreliable as a routine diagnostic method. Furthermore, AR-Manual assessments showed a notable bias for higher values than AR-DIA. This discrepancy was caused by weak nuclear or strong cytoplasmic staining, which should be considered as a false positive as it has no prognostic impact; meanwhile, the DIA method did not detect this faint false-positive staining. As we have shown before, strict standardization and objective quantitative digital measurements provide more reproducible and prognostically robust results [37,38,39]. Once AR-DIA is fully validated and developed, it could improve accuracy and precision, eliminate subjectivity, and save considerable time. However, many laboratories do not have DIA technology and must rely on manual, subjective AR quantification. In this situation, the following criteria should be considered to improve the precision and reproducibility of AR assessment: if the tumor grade is 1 or 2, or if the tumor has MAI < 5, the AR assessment should be repeated, as these cases often show AR ≥ 10%. Additionally, for cases with AR expression between 10% and 50%, the measurement should be repeated by a second observer, and the inter-observer variability should be assessed. A simple point-sampling method for subjective AR measurement may also be helpful.

In ER-positive breast cancer, AR functions as a tumor suppressor by inhibiting cell proliferation and interfering with ER signaling via competitive binding to the estrogen response element—a concept supported by a meta-analysis [40]. Additionally, research has shown that AR plays a role in regulating the proliferation, migration, invasion, and growth of ER-negative breast cancer cells in vivo [41]. AR has different signaling pathways that contribute to breast cancer pathogenesis. The luminal androgen receptor (LAR) subtype of TNBC, which is characterized by high AR expression, shares some molecular features with luminal-type breast cancers [42,43,44]. This subtype generally exhibits lower proliferation and lower grades, consistent with AR’s inhibitory effects. However, in later-stage patients, high AR expression may shift roles and act as an oncogene, driving processes such as invasion, proliferation, and migration. We identified dual, opposing prognostic roles of AR in TNBC. High AR-DIA was significantly associated with low grade and low proliferation, but it was also correlated with larger tumor size, distant metastasis, and LN positivity. These contrasting observations may help to explain the controversy surrounding reports on the prognostic and predictive value of high AR in TNBC. Another factor contributing to these conflicting results could be the composition of patient cohorts, as population-specific patterns have been reported, with cohorts in the U.S. and Nigeria showing opposite results to those from Ireland, India, and Norway [18].

A recent meta-analysis of 27 studies, including 4914 TNBC patients, found no significant relationship between AR expression and survival [22]. However, this meta-analysis did not differentiate between LN-negative and LN-positive patients, nor did it consider distinct populations despite the wide range of AR positivity. Another previous study from Norway (*n* = 37) [17] reported no impact of AR expression on prognosis in TNBC. However, tissue microarrays (TMAs) were utilized, which may have caused sampling errors due to tumor heterogeneity, contributing to the failure to detect a prognostic effect of AR expression. Supporting this, one study actually assessed AR expression using both TMAs and WSIs and showed a prognostic effect with WSIs but not with TMAs [45]. To date, high AR expression has been identified as a marker of worse prognosis in some studies [15,18,20], including ours, while other studies have shown it to be associated with better prognosis [13,16,18]. These variations are likely attributed to cohort characteristics, tumor sampling methods, length of follow-up, laboratory procedures, assay methods, and differences in cut-off values. Further research is needed to evaluate TNBC cases across multiple institutions in Norway, in order to establish a definitive national cut-off value and better assess the prognostic implications of AR expression.

Analyses grouped by different combinations of LN status, MAI, FF, sTILs, and AR-DIA showed that ACT had a positive impact on survival in some of the unfavorable low AR-DIA subgroups, but not in patients with high AR-DIA. High sTILs demonstrated positive prognostic significance, as previously observed in other studies [28,46,47,48], and sTILs have opened new approaches for treatment decisions in TNBC. Given the ongoing search for treatment options beyond chemotherapy in TNBC, the high AR observed in patients with low sTILs—who lack the option for immunotherapy—highlights the potential importance of anti-androgen therapy for this subgroup.

Although the prognostic impact of AR in TNBC patients is still controversial, the role of AR is becoming increasingly important [23]. AR is recognized as an oncogenic driver of TNBC, and AR-positive TNBC patients may benefit from targeted treatments against AR [42], which have already been implemented in the treatment of prostate cancer [49]. Understanding the specific molecular subtypes of TNBC is crucial for developing targeted and effective treatment strategies, due to the dual opposing effect. Clinical trials in TNBC patients with high AR, investigating AR-targeted therapies such as bicalutamide, enzalutamide, and darolutamide, are planned or underway. The combination of AR-targeted agents with other treatments, alongside monotherapy, theoretically offers intriguing new strategies. Combination with drugs that target CDK4/6, CYP17A1, or the PI3K/AKT/mTOR pathway, immunotherapies, or conventional treatments such as chemotherapy and radiotherapy may enhance the efficacy of breast cancer treatment [50,51]. This approach may warrant further investigation for its clinical effectiveness, and several clinical trials are currently exploring AR-targeted therapy as both monotherapy and combination therapy. Additionally, the interaction between LN status and AR-DIA identified in the multivariate analysis was noteworthy. High AR-DIA in LN-positive patients was an indicator of a worse prognosis, suggesting that AR-targeted therapy should be considered. These two factors should be evaluated together, which could provide a more personalized approach to treatment selection.

The strengths of this study are the evaluation of a population-based cohort with long-term follow-up, as well as its use in standardized fixation and tissue processing, sectioning, IHC, and DIA measurement methods. However, there are some limitations to this study. All patient data were obtained from a single institution, which may limit the generalizability of the findings. Additionally, some of the tumor tissues used in the study were old. Although paraffin blocks are known to preserve antigenicity over many years [52], the potential loss of these properties over time should be considered. Moreover, the follow-up time in survival analyses was limited to the first 15 years. Since distant metastases were detected in only 2 cases after 15 years, it is believed that sufficient data may not have been available in these long-term follow-ups. In addition, the small sample sizes in the subgroups may reduce the power of the analyses and lead to overinterpretation. Therefore, similar studies in a larger, more balanced, and standardized multi-institutional context should be conducted to strengthen the results and minimize the impact of bias and random variability. Finally, the ACT administered to patients may have varied over the years, as the Norwegian treatment guidelines have evolved in terms of agents and dosing frequency over time. For example, some of the cases were from earlier years, and so, some of these patients did not receive treatment. However, with the discovery of new agents and biomarkers, treatment options have increased. This variability should be considered when interpreting the ACT effect analysis results.

In conclusion, identifying AR in TNBC can add crucial information on the prognosis of patients and choosing patients that can be considered for AR-targeted therapies. The evaluation of AR using DIA should be more widely conducted in routine practice, as it can improve reproducibility while reducing subjectivity. AR expression in ≥10% of tumor cells assessed by means of DIA was found to be an independent predictor of worse prognosis in TNBC. AR should be evaluated alongside LN status, as their combination was found to have the strongest negative effect on survival. Particularly in unfavorable subgroups with high AR expression, patients could benefit from AR-targeted therapy. This study also revealed that high AR-DIA in TNBCs has a dual opposing function, which may explain the variable prognostic outcomes of AR reported in different studies and the therapeutic heterogeneity of TNBC.

## Figures and Tables

**Table 1:** Cross-tabulation of AR-Manual and AR-DIA with a 10% threshold (*p* < 0.001).

	AR-Manual		
AR-DIA	<10%	≥10%	Total
<10%	81 (100%)	16 (14%)	97 (49%)
≥10%	0 (0%)	101 (86%)	101 (51%)
Total	81 (41%)	117 (59%)	198

AR: androgen receptor; AR-DIA: androgen receptor digital image analysis.

**Table 2:** Correlation between the clinicopathological characteristics and AR expression by manual and DIA counting.

Characteristics	Total	AR-Manual		AR-DIA	
		<10%	≥10%	<10%	≥10%
Age (Years)					
<50	75	32 (42.7)	43 (57.3)	39 (52.0)	36 (48.0)
≥50	123	49 (39.8)	74 (60.2)	58 (47.2)	65 (52.8)
*p*-Value *OR, CI 95%*		0.694 n.s.		0.559 n.s.	
Tumor Size (cm)					
<1	11	6 (54.5)	5 (45.5)	6 (54.5)	5 (45.5)
1–1.9	67	34 (50.7)	33 (49.3)	41 (61.2)	26 (38.8)
2–2.9	54	16 (29.6)	38 (70.4)	20 (37.0)	34 (63.0)
≥3	64	23 (35.9)	41 (64.1)	28 (43.8)	36 (56.3)
*p*-Value *OR, CI 95%*		0.071 n.s.		0.047 0.76, 0.21–2.74 2.04, 0.55–7.55 1.54, 0.42–5.58	
Nottingham Grade					
Grade 1	11	2 (18.2)	9 (81.8)	3 (27.3)	8 (72.7)
Grade 2	37	11 (29.7)	26 (70.3)	13 (35.1)	24 (64.9)
Grade 3	150	68 (45.3)	82 (54.7)	81 (54.0)	69 (46.0)
*p*-Value *OR, CI 95%*		0.065 n.s.		0.038 0.69, 0.15–3.06 0.31, 0.82–1.25	
Nottingham Grade					
Grade 1 + 2	48	13 (27.1)	35 (72.9)	16 (33.3)	32 (66.7)
Grade 3	150	68 (45.3)	82 (54.7)	81 (54.0)	69 (46.0)
*p*-Value *OR, CI 95%*		0.029 0.44, 0.22–0.91		0.014 0.42, 0.21–0.84	
Histologic Type					
NST	169	68 (40.2)	101 (59.8)	84 (49.7)	85 (50.3)
Others	29	13 (44.8)	16 (55.2)	13 (44.8)	16 (55.2)
*p*-Value *OR, CI 95%*		0.642 n.s.		0.627 n.s.	
MAI5					
<5	25	4 (16.0)	25 (84.0)	5 (20.0)	20 (80.0)
≥5	173	77 (44.5)	96 (55.5)	92 (53.2)	81 (46.8)
*p*-Value *OR, CI 95%*		0.007 0.23, 0.78–0.72		0.004 0.22, 0.07–0.61	
MAI10					
<10	59	12 (20.3)	47 (79.7)	15 (25.4)	44 (74.6)
≥10	139	69 (49.6)	70 (50.4)	82 (59.0)	57 (41.0)
*p*-Value *OR, CI 95%*		<0.001 0.25, 0.12–0.53		<0.001 0.23, 0.12–0.45	
sTILs (%)					
<40	145	58 (40.0)	87 (60.0)	65 (44.8)	80 (55.2)
≥40	53	23 (43.4)	30 (56.6)	32 (60.4)	21 (39.6)
*p*-Value *OR, CI 95%*		0.745 n.s.		0.056 n.s.	
Fibrotic Focus					
Absent	108	43 (39.8)	65 (60.2)	50 (46.3)	58 (53.7)
Present	90	38 (42.2)	52 (57.8)	47 (52.2)	43 (47.8)
*p*-Value *OR, CI 95%*		0.773 n.s.		0.476 n.s.	
Lymph Node Status					
Negative	103	54 (52.4)	49 (47.6)	64 (62.1)	39 (37.9)
Positive	95	27 (28.4)	68 (71.6)	33 (34.7)	62 (65.3)
*p*-Value *OR, CI 95%*		0.001 2.77, 1.53–5.00		<0.001 3.08, 1.72–5.50	
Distant Metastasis (DM)					
Non-DM	129	57 (44.2)	72 (55.8)	70 (54.3)	59 (45.7)
DM	69	24 (34.8)	45 (65.2)	27 (39.1)	42 (60.9)
*p*-Value *OR, CI 95%*		0.227 n.s.		0.052 1.84, 1.01–3.34	

AR: androgen receptor, DIA: digital image analysis, *p*-value: probability of no significant difference, OR: odds ratio, CI 95%: 95% confidence interval, MAI: mitotic activity index, sTILs: stromal tumor-infiltrating lymphocytes; n.s.: non-significant.

**Table 3:** The univariate survival analysis of all characteristics in the 15-year follow-up.

Characteristics		Events/At Risk (DMFS %)	Log Rank *p*-Value	Hazard Ratio	95% Confidence Interval
Age (Years)	<50	25/75 (67)	0.266	n.s.	n.s.
	≥50	44/123 (64)			
Tumor Size (cm)	<1	1/11 (91)	0.180	n.s.	n.s.
	1–1.9	24/67 (64)			
	2–2.9	17/54 (68)			
	≥3	27/64 (58)			
Nottingham Grade	Grade 1	3/11 (73)	0.089	n.s.	n.s.
	Grade 2	8/37 (78)			
	Grade 3	58/150 (61)			
Nottingham Grade	Grade 1 + 2	11/48 (77)	0.029	2.02	1.06–3.85
	Grade 3	58/150 (61)			
Histologic Type	NST	61/169 (64)	0.287	n.s.	n.s.
	Others	8/29 (72)			
MAI5	<5	3/25 (88)	0.015	3.81	1.19–12.12
	≥5	66/173 (62)			
MAI10	<10	17/59 (71)	0.263	n.s.	n.s.
	≥10	52/139 (63)			
sTILs (%)	<40	58/145 (60)	0.012	0.44	0.23–0.85
	≥40	11/53 (79)			
Fibrotic Focus (FF)	Absent	27/108 (75)	0.001	2.21	1.36–3.59
	Present	42/90 (53)			
AR-Manual (%)	<10	24/81 (70)	0.065	n.s.	n.s.
	≥10	45/117 (61)			
AR-DIA (%)	<10	27/97 (72)	0.008	1.91	1.17–3.10
	≥10	42/101 (58)			
Lymph Node Status	Negative	24/103 (77)	<0.001	2.82	1.71–4.65
	Positive	45/95 (53)			
Treatment	No Chemotherapy	22/62 (64)	0.978	n.s.	n.s.
	Chemotherapy	42/113 (63)			

DMFS: distant metastasis-free survival, *p*-value: probability of no significant difference, MAI: mitotic activity index, sTILs: stromal tumor-infiltrating lymphocytes, AR: androgen receptor, DIA: digital image analysis; n.s.: non-significant.

**Table 4:** Multivariate Cox regression analysis (final model) of the most significant features and interactions between features, developed using Forward Stepwise (Likelihood Ratio) selection (chi-square = 45.578, *p* < 0.001).

Features	Beta	Standard Error Beta	Wald	*p*-Value	Hazard Ratio	95% CI
LN and AR-DIA interaction	0.49	0.099	24.6	<0.001	1.63	1.34–1.98
FF	0.65	0.251	6.8	0.009	1.93	1.18–3.16
MAI5	1.41	0.598	5.5	0.018	4.10	1.27–13.23
sTILs40	−0.67	0.331	4.1	0.041	0.50	0.26–0.97

*p*-value: probability of no significant difference, LN: lymph node, AR-DIA: androgen receptor digital image analysis, FF: fibrotic focus, MAI: mitosis activity index, sTILs: stromal tumor-infiltrating lymphocytes; CI: confidence interval of the hazard ratio.

**Table 5:** Prognostic evaluation of AR-DIA in unfavorable TNBC subgroups.

Characteristics of Subgroup	AR-DIA (%)	Events/At Risk (DMFS %)	Log Rank *p*-Value	Hazard Ratio	95% Confidence Interval
LN Pos	<10	12/33 (64)	0.026	2.09	1.07–4.06
	≥10	33/62 (47)			
MAI ≥ 5	<10	26/92 (72)	<0.001	2.38	1.45–3.91
	≥10	40/81 (51)			
FF-Present	<10	17/47 (64)	0.007	2.30	1.23–4.29
	≥10	25/43 (42)			
sTILs < 40	<10	22/65 (66)	0.047	2.21	0.67–7.26
	≥10	36/80 (55)			
LN Pos, MAI ≥ 5	<10	11/31 (65)	0.009	2.43	1.21–4.86
	≥10	31/53 (42)			
LN Pos, FF-Present	<10	6/18 (67)	0.004	3.62	1.42–9.19
	≥10	19/28 (32)			
LN Pos, sTILs < 40	<10	8/21 (62)	0.034	2.29	1.04–5.03
	≥10	29/50 (42)			
LN Pos, MAI ≥ 5, FF-Present	<10	6/18 (67)	0.003	3.87	1.51–9.90
	≥10	18/25 (28)			
LN Pos, MAI ≥ 5, sTILs < 40	<10	7/20 (65)	0.010	2.86	1.23–6.60
	≥10	27/42 (36)			
LN Pos, MAI ≥ 5, FF-Present, sTILs < 40	<10	4/12 (67)	0.004	4.58	1.49–14.05
	≥10	15/20 (25)			
LN Pos, FF-Present, sTILs < 40	<10	4/12 (67)	0.006	4.17	1.37–12.66
	≥10	16/23 (30)			
MAI ≥ 5, FF-Present	<10	17/47 (64)	0.002	2.60	1.38–4.88
	≥10	24/38 (37)			
MAI ≥ 5, sTILs < 40	<10	21/61 (66)	0.005	2.15	1.24–3.71
	≥10	34/63 (46)			
MAI ≥ 5, FF-Present, sTILs < 40	<10	15/37 (60)	0.006	2.58	1.29–5.16
	≥10	19/29 (35)			
FF-Present, sTILs < 40	<10	15/37 (60)	0.021	2.17	1.10–4.27
	≥10	20/34 (41)			

AR: androgen receptor, DIA: digital image analysis, DMFS: distant metastasis-free survival, *p*-value: probability of no significant difference, LN: lymph node, pos: positive, MAI: mitotic activity index, FF: fibrotic focus; sTILs: stromal tumor-infiltrating lymphocytes.

## Data Availability

The dataset generated during the current study is not publicly available because of ethical and legal concerns. Anonymized data can be made available from the Stavanger University Hospital Institutional Data Access/Ethics Committee (contact via email: rek-vest@uib.no, REK vest, Rogaland, Vestland, Norway) for researchers who meet the criteria for access to confidential data.

## Supplementary Materials

The following supporting information can be downloaded at https://www.mdpi.com/article/10.3390/bioengineering12010054/s1: Figure S1: Median scores for AR-Manual (30%) and AR-DIA (11%) (**a**), median AR-DIA scores for LN-positive (23%) and LN-negative (3%) patients (**b**); Table S1: Summary of performance metrics under different AR-DIA thresholds; Table S2: The univariate survival analysis of all characteristics in the long-term follow-up; Table S3: Evaluation of AR-DIA < 10% patients with consideration of the following aspects: number of patients, total number of patients, distant metastasis-free survival, *p*-value, hazard ratio, and 95% confidence interval. The secondary considerations were sensitivity, specificity, negative predictive value, positive predictive value, and overall correct percentage.
