# Supplementary material for: Dual Functions of Androgen Receptor Overexpression in Triple-Negative Breast Cancer: A Complex Prognostic Marker
Source: Bioengineering (Basel). 2025 Jan 10;12(1):54. doi: 10.3390/bioengineering12010054 (PMC11761274; doi:10.3390/bioengineering12010054)
Supplement: Supplementary file 1 [file bioengineering-12-00054-s001.zip › Supplementary Table S1.pdf]

Supplementary file

Dual Functions of Androgen Receptor Overexpression in Triple-Negative Breast Cancer: A Complex Prognostic Marker

Umay Kiraz[1,2*], Emma Rewcastle[1], Silja Kavlie Fykse[1], Ingrid Lundal[1], Einar G. Gudlaugsson[1], Ivar Skaland[1], Håvard Søiland[#,4], Jan P. A. Baak[1,†], Emiel A. M. Janssen[1,2,3,†]

[1] Department of Pathology, Stavanger University Hospital, 4011 Stavanger, Norway

[2] Department of Chemistry, Bioscience and Environmental Engineering, University of Stavanger, 4021 Stavanger, Norway

[3] Institute for Biomedicine and Glycomics, Griffith University, Queensland, Australia.

[4] Department of Research, Stavanger University Hospital, Stavanger, Norway

*Correspondence: umaykiraz@gmail.com, ORCID: 0000-0002-6721-4877

† These authors contributed equally to this work

# Prof. Håvard Søiland passed away before the proofreading of the article. This article is dedicated to his continuous fight against breast cancer.

Table S1 Summary of performance metrics under different AR-DIA thresholds.

AR-DIA	Chi-value	*p*-Value	n events under threshold	n censored under threshold	n total under threshold (%)	Specificity	Sensitivity	Negative Predictive Values	Positive Predictive Values	% Correct
**1%**	1.3	0.249	20	43	63 (10%)	43/129 (33%)	49/69 (71%)	43/63 (68%)	49/135 (36%)	92/198 (46%)
**5%**	4.5	0.034	26	63	89 (45%)	63/129 (49%)	43/69 (63%)	63/89 (71%)	43/109 (39%)	106/198 (53%)
**10%**	7.1	0.008	27	70	97 (49%)	70/129 (54%)	42/69 (61%)	70/97 (72%)	42/101 (41%)	112/198 (56%)
**15%**	2.6	0.104	35	74	109 (55%)	74/129 (57%)	34/69 (49%)	74/109 (68%)	34/89 (38%)	108/198 (54%)
**20%**	2.6	0.106	37	78	115 (58%)	78/129 (60%)	32/69 (46%)	78/115 (68%)	32/83 (38%)	110/198 (55%)

AR-DIA: androgen receptor-digital image analysis, *p*-value: probability of no significant difference, n: number.
