# Supplementary material for: Dual Functions of Androgen Receptor Overexpression in Triple-Negative Breast Cancer: A Complex Prognostic Marker
Source: Bioengineering (Basel). 2025 Jan 10;12(1):54. doi: 10.3390/bioengineering12010054 (PMC11761274; doi:10.3390/bioengineering12010054)
Supplement: Supplementary file 1 [file bioengineering-12-00054-s001.zip › Supplementary Figure S1.pdf]

Supplementary file

Dual Functions of Androgen Receptor Overexpression in Triple-Negative Breast Cancer: A Complex Prognostic Marker

Umay Kiraz[1,2*], Emma Rewcastle[1], Silja Kavlie Fykse[1], Ingrid Lundal[1], Einar G. Gudlaugsson[1], Ivar Skaland[1], Håvard Søiland[#,4], Jan P. A. Baak[1,†], Emiel A. M. Janssen[1,2,3,†]

[1] Department of Pathology, Stavanger University Hospital, 4011 Stavanger, Norway

[2] Department of Chemistry, Bioscience and Environmental Engineering, University of Stavanger, 4021 Stavanger, Norway

[3] Institute for Biomedicine and Glycomics, Griffith University, Queensland, Australia.

[4] Department of Research, Stavanger University Hospital, Stavanger, Norway

*Correspondence: umaykiraz@gmail.com, ORCID: 0000-0002-6721-4877

† These authors contributed equally to this work

# Prof. Håvard Søiland passed away before the proofreading of the article. This article is dedicated to his continuous fight against breast cancer.

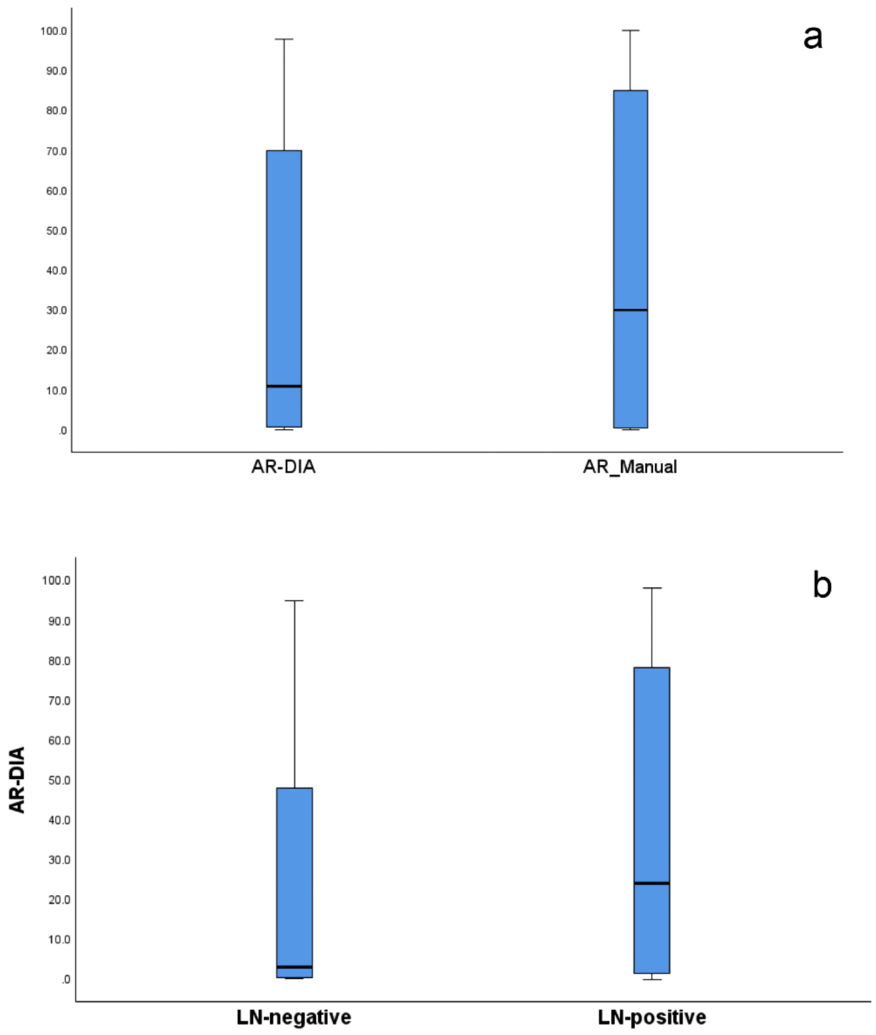

Figure S1. Median scores for AR-Manual (30%) and AR-DIA (11%) (**a**), median AR-DIA scores for LN-positive (23%) and LN-negative (3%) patients (**b**).
