# Supplementary material for: Dual Functions of Androgen Receptor Overexpression in Triple-Negative Breast Cancer: A Complex Prognostic Marker
Source: Bioengineering (Basel). 2025 Jan 10;12(1):54. doi: 10.3390/bioengineering12010054 (PMC11761274; doi:10.3390/bioengineering12010054)
Supplement: Supplementary file 1 [file bioengineering-12-00054-s001.zip › Supplementary Table S3.pdf]

Supplementary file

Dual Functions of Androgen Receptor Overexpression in Triple-Negative Breast Cancer: A Complex Prognostic Marker

Umay Kiraz[1,2*], Emma Rewcastle[1], Silja Kavlie Fykse[1], Ingrid Lundal[1], Einar G. Gudlaugsson[1], Ivar Skaland[1], Håvard Søiland[#,4], Jan P. A. Baak[1,†], Emiel A. M. Janssen[1,2,3,†]

[1] Department of Pathology, Stavanger University Hospital, 4011 Stavanger, Norway

[2] Department of Chemistry, Bioscience and Environmental Engineering, University of Stavanger, 4021 Stavanger, Norway

[3] Institute for Biomedicine and Glycomics, Griffith University, Queensland, Australia.

[4] Department of Research, Stavanger University Hospital, Stavanger, Norway

*Correspondence: umaykiraz@gmail.com, ORCID: 0000-0002-6721-4877

† These authors contributed equally to this work

# Prof. Håvard Søiland passed away before the proofreading of the article. This article is dedicated to his continuous fight against breast cancer.

Table S3. Evaluation of AR-DIA<10% patients with consideration of the following aspects: number of patients, total number of patients, distant metastasis-free survival, P-value, hazard ratio, and 95% confidence interval. The secondary considerations were sensitivity, specificity, negative predictive value, positive predictive value, and overall correct percentage.

	number	number in total	Survival %	*p*-Value	HR	95% CI	Sensitivity %	Specifity %	NPV %	PPV %	% correct
**LNpos, FF present, AR-DIA<10**											
ACT	16	18	75	0.023	6.260	1.02–38.13	67	0	0	25	22
NonACT	2		0								
**LNpos, MAI≥5, FF present, AR-DIA<10**											
ACT	16	18	75	0.023	6.260	1.02–38.19	67	0	0	25	22
NonACT	2		0								
**LNpos, FF present, sTILs<40, AR-DIA<10**											
ACT	10	12	80	0.012	11.916	1.05–134.90	50	0	0	20	17
NonACT	2		0								
**LNpos, MAI≥5, FF present, sTILs<40, AR-DIA<10**											
ACT	10	12	80	0.012	11.916	1.05–134.90	50	0	0	20	17
NonACT	2		0								

p-value: probability of no significant difference, HR: hazard ratio, CI: confidence interval of the hazard ratio, NPV: negative predictive value, PPV: positive predictive value, LN: lymph node, pos: positive, ACT: adjuvant chemotherapy, nonACT: non-adjuvant chemotherapy, MAI: mitotic activity index, FF: fibrotic focus, sTILs: stromal tumor-infiltrating lymphocytes, AR: androgen receptor, DIA: digital image analysis.
