# Supplementary material for: Dual Functions of Androgen Receptor Overexpression in Triple-Negative Breast Cancer: A Complex Prognostic Marker
Source: Bioengineering (Basel). 2025 Jan 10;12(1):54. doi: 10.3390/bioengineering12010054 (PMC11761274; doi:10.3390/bioengineering12010054)
Supplement: Supplementary file 1 [file bioengineering-12-00054-s001.zip › Supplementary Table S2.pdf]

Supplementary file

Dual Functions of Androgen Receptor Overexpression in Triple-Negative Breast Cancer: A Complex Prognostic Marker

Umay Kiraz[1,2*], Emma Rewcastle[1], Silja Kavlie Fykse[1], Ingrid Lundal[1], Einar G. Gudlaugsson[1], Ivar Skaland[1], Håvard Søiland[#,4], Jan P. A. Baak[1,†], Emiel A. M. Janssen[1,2,3,†]

[1] Department of Pathology, Stavanger University Hospital, 4011 Stavanger, Norway

[2] Department of Chemistry, Bioscience and Environmental Engineering, University of Stavanger, 4021 Stavanger, Norway

[3] Institute for Biomedicine and Glycomics, Griffith University, Queensland, Australia.

[4] Department of Research, Stavanger University Hospital, Stavanger, Norway

*Correspondence: umaykiraz@gmail.com, ORCID: 0000-0002-6721-4877

† These authors contributed equally to this work

# Prof. Håvard Søiland passed away before the proofreading of the article. This article is dedicated to his continuous fight against breast cancer.

Table S2. The univariate survival analysis of all characteristics in the long-term follow-up.

Characteristics		Events / At Risk (DMFS %)	Log Rank *p*-Value	Hazard Ratio	95% Confidence Interval
Age (years)	<50	25 / 75 (67)	0.154	n.s.	n.s.
	≥50	44 / 123 (64)			
Tumor size (cm)	<1	1 / 11 (91)	0.142	n.s.	n.s.
	1-1.9	24 / 67 (64)			
	2-2.9	17 / 54 (68)			
	≥3	27 / 64 (58)			
Nottingham Grade	Grade 1	3 / 11 (73)	0.104	n.s.	n.s.
	Grade 2	8 / 37 (78)			
	Grade 3	58 / 150 (61)			
Nottingham Grade	Grade 1+2	11 / 48 (77)	0.035	1.98	1.04–3.78
	Grade 3	58 / 150 (61)			
Histologic type	NST	61 / 169 (64)	0.317	n.s.	n.s.
	Others	8 / 29 (72)			
MAI5	<5	3 / 25 (88)	0.015	3.81	1.20–12.15
	≥5	66 / 173 (62)			
MAI10	<10	17 / 59 (71)	0.285	n.s.	n.s.
	≥10	52 / 139 (63)			
sTILs (%)	<40	58 / 145 (60)	0.010	0.44	0.23–0.83
	≥40	11 / 53 (79)			
Fibrotic focus (FF)	Absent	27 / 108 (75)	<0.001	2.21	1.36–3.59
	Present	42 / 90 (53)			
AR-Manual (%)	<10	24 / 81 (70)	0.111	n.s.	n.s.
	≥10	45 / 117 (61)			
AR-DIA (%)	<10	27 / 97 (72)	0.016	1.80	1.11–2.93
	≥10	42 / 101 (58)			
Lymph Node Status	Negative	24 / 103 (77)	<0.001	2.63	1.60–4.33
	Positive	45 / 95 (53)			
Treatment	No chemotherapy	22 / 62 (64)	0.968	n.s.	n.s.
	Chemotherapy	42 / 113 (63)			

DMFS: distant metastasis-free survival, *p*-value: probabilities of no significant differences, MAI: mitotic activity index, sTILs: stromal tumor-infiltrating lymphocytes, AR: androgen receptor, DIA: digital image analysis, n.s.: non-significant.
